# Supplementary material for: Assessment of publication bias and outcome reporting bias in systematic reviews of health services and delivery research: A meta-epidemiological study
Source: PLoS One. 2020 Jan 30;15(1):e0227580. doi: 10.1371/journal.pone.0227580 (PMC6992172; doi:10.1371/journal.pone.0227580)
Supplement: Attachment — Submitted filename: Response to reviewers.docx [file pone.0227580.s001.docx]

We appreciate the editor and reviewers for the informative comments provided on this manuscript. Please find our responses to each peer reviewers’ comments, in red, below.

**Reviewer 1**

Reviewer #1: I wish to thank the editors for the opportunity to review this manuscript entitled “Assessment of publication bias and outcome reporting bias in systematic reviews of health services and delivery research: a meta-epidemiological study”. The authors have assessed how often publication bias and selective outcome reporting bias was mentioned and assessed in a random sample of systematic reviews of studies in the field of health services and delivery research. They have explained and reported their methods transparently.

I have the following comments for the authors:
1. Ll. 72-73: I suggest the authors explain in more detail why biased results of systematic reviews are a problem. E.g., as stated in their PROSPERO record: “This is important because HSDR frequently informs decisions at institutional and policy levels, and failure to recognise bias in evidence used to inform decisions could have substantial implications for population health and resource allocation.”

We have included a sentence “In HSDR, this could have substantial implications for population health and resource allocation”

2. Ll. 97-116: I feel this part belongs to the method section and recommend to incorporate it in “eligibility criteria”.

We have introduced these texts early in the manuscript as we believe that a clear description of the concepts of intervention and association reviews is necessary for us to explain the rationale and focus of the study, which are better located in the Introduction section.

3. Ll. 100-104 (“We examined key features”): In my view, this is repetitive and thus can be deleted.

The sentence has now been deleted.

4. Ll. 104-105: I am surprised that the study was registered in PROSPERO as it does not contain a health-related outcome. Nevertheless, I consider it very valuable that the methods have been pre-specified. I suggest that the authors delete the term “protocol” (because strictly speaking it is a registration record) and encourage them to update their PROSPERO record’s status to “completed but not published” (and remind to update it again to “completed and published” once their manuscript has been published). When doing so, they may want to explain important changes to their record since its initial version, i.e. the addition of two new authors, the changes in the search strategy already outlined in this manuscript, and the assessment with AMSTAR.

We have deleted the term ‘protocol’ and changed the wording to “The review was pre-registered”. We have also changed the term “registered protocol” in the following section (Sampling strategy) to “PROSPERO registration record”. We have submitted an update for the PROSPERO record as suggested by the reviewer.

5. Ll. 137 and following (“Eligibility criteria”): I suggest to add the following information (taken from the PROSPERO record) at the end of this sub section: “Where more than one review within the initially selected samples cover overlapping interventions or associations, only the latest review will be retained to maintain the independence of observations (i.e. reduce overlap of included studies between reviews) and to capture the contemporary practice.” The authors should discuss the issue of potentially remaining overlap in the limitations section.

In the eligibility criteria section we have added a sentence “Where more than one review within the initially selected samples cover overlapping interventions or associations, we included the latest review. This helped to maintain the independence of observations and also capture the contemporary practice.”

In the discussion of limitations we have included “We ensured that we only include the latest systematic review whenever we identified more than one which covers overlapping topics. However, there may be some overlap in the studies included within different systematic reviews but we do not believe this would have significant impact on our findings as our study focuses on the overall features and methodology of the sampled systematic reviews rather than individual studies included within them.”

6. LL. 181-197 (data items): In their PROSPERO record, the authors stated that they would categorize the reviews according to the types of journal they were published in (medical journals, health services research and health policy journals, management and social science journals or others (including grey literature)). If that has been done, I suggest to add it as a data item and in the results. If not, the authors may want to describe that in the revision note for their updated PROSPERO record.

We performed preliminary classification as planned but discovered substantial overlap in the features between journal types, which hindered reliable classification of some journals and in turn could cause difficulty in the interpretation of any comparisons made based on the classification. We discussed our observation with the study steering committee members, who suggested us to use journal endorsement of systematic review guidelines and journal impact factors to characterise the journals instead.

We have now described these in the Methods section, and have highlighted this on the updated PROSPERO record.

7. Ll. 213-214: If possible, the authors should explain in more detail the methodology that was used by HSE to assess the systematic reviews with AMSTAR (i.e. two people independently?). Furthermore, the authors should discuss in the limitations that the tool has several weaknesses (which eventually lead to the development of a new version of the tool <https://www.bmj.com/content/bmj/358/bmj.j4008.full.pdf> ).

We have clarified that the quality assessment of included systematic reviews done by the HSE were conducted independently by two reviewers.

We have now also discussed AMSTAR in the ‘Strengths and limitations’ section of the Discussion: “HSE used AMSTAR to assess the quality of included systematic reviews. We have also used the same tool to assess the quality of the five systematic reviews with missing AMSTAR scores in order to maintain consistency. However, AMSTAR was designed for quality assessment of systematic reviews of RCTs of interventions and therefore some of the items were not relevant for many of the systematic reviews in this study. An updated version of the tool, AMSTAR 2, was published in 2017 which includes items relevant to non-randomised studies and would have been more appropriate to assess the qualities of the systematic reviews included in this study”.

8. Table 1: I was wondering if the authors have extracted the studies’ publication year. I consider this an important characteristic and strongly suggest that the authors add this in their analyses. For example, if it turned out that the association reviews were all published in 2007-2009 (before PRISMA was published), while most intervention reviews were published after 2009 (after PRISMA was published), this might also explain why fewer of them mentioned publication bias.

Yes, we have data on year of publication. There is no major difference in the type of review by year of publication with regards to the period before versus after PRISMA’s publication. There were 19% of association reviews and 16% of intervention reviews published before PRISMA. Even when only studies published after 2009 were considered, a higher proportion of intervention reviews mentioned publication bias; 58% (n=59/84) of intervention reviews and 33% (n=27/81) of association reviews mentioned publication bias. We therefore believe that publication before/after PRISMA do not account for the differences observed in association and intervention reviews.

9. Table 1: “AMSTAR rating” should be specified, e.g. “Percentage of AMSTAR items rated positively” and “Journal endorses systematic review guideline” should be complemented by “(as of 2018)”

We have revised the texts as suggested.

10. Ll. 273: Please quantify “often”, i.e. in how many review it was reported that the conditions for using funnel plots were not met?

This is related to five cases where reviewers have reported planning to assess publication bias but not able to do so. We have reworded this sentence to reflect this more clearly and included the references for these cases. “In five of the systematic reviews, authors reported planning statistical assessment of publication bias but did not carry out the assessment due to conditions of using funnel plots not being met, especially insufficient number of studies and/or heterogeneity between included studies.[20-24]”

This also applies to ll. 323; please specify in how many reviews the lack of protocols was reported as a barrier to assessing outcome reporting bias. It may be worth to draw up a table that includes all reasons that systematic review authors named as barriers to assessing publication bias or outcome reporting bias.

As highlighted in the manuscript, all studies which mentioned outcome reporting bias assessed it. This sentence in line 323 of the original manuscript refers to the reasons why reviewers have judged some included studies as “unclear” when assessing outcome reporting bias. Generally, ‘unclear’ indicates lack of information to make necessary judgement. We have reworded the sentence to make it clearer and indicated the number of reviews where this was specifically mentioned with references. “In three reviews, lack of pre-registered protocols reported as a reason for judging articles as ‘unclear’.[19, 21, 27] In a review in which the review authors explicitly stated that they did not search for study protocols, 13 out of the 19 included studies were judged as ‘unclear’ on selective outcome reporting.[28]”

11. Ll. 287 and 306: Commas are missing after the odds ratios.

Thank you. We have inserted commas.

12. Ll. 363 and following (Strengths and limitations): In addition to issues mentioned before, the authors should discuss that the item “endorsement of reporting guidelines” was assessed as of 2018, but that it is unclear if it has been the case at the time the systematic review authors have prepared their manuscripts. Furthermore, the authors should discuss that reporting guidance (at least by Cochrane and PRISMA) is aimed at systematic reviews of interventions. So, authors of systematic reviews on associations may not have followed it even if it was endorsed by the journal they have published in.

We have included “The association between journal’s endorsement and authors’ use of reporting guidelines and assessment of publication bias may not have been characterised very accurately in our study. We classified journals based on endorsement of reporting guidelines as of 2018 but we were not able to determine if this has been the case as at the time the systematic review authors prepared/published their manuscripts. Notwithstanding, journal endorsement of such guidelines may be an indication of the journal’s generic requirement of higher standard of reporting. Also, available reporting guidelines are mostly aimed at systematic reviews of intervention studies and authors of systematic reviews of association studies might not have considered that it was necessary to follow such guidelines, even if it was endorsed by the journal they published in. Alternatively, some authors might have used reporting guidelines during the preparation of their reviews without explicitly stating it.”

13. Ll. 367: Please provide citations when referring to “previous studies”.

We have included three citations:

Ziai H, Zhang R, Chan A-W, Persaud N. Search for unpublished data by systematic reviewers: an audit. 2017;7(10). doi: 10.1136/bmjopen-2017-017737 %J BMJ Open.

Herrmann D, Sinnett P, Holmes J, Khan S, Koller C, Vassar M. Statistical controversies in clinical research: publication bias evaluations are not routinely conducted in clinical oncology systematic reviews. Annals of oncology : official journal of the European Society for Medical Oncology. 2017;28(5):931-7. Epub 2017/01/01. doi: 10.1093/annonc/mdw691. PubMed PMID: 28039176.

Chapman SJ, Drake TM, Bolton WS, Barnard J, Bhangu A. Longitudinal analysis of reporting and quality of systematic reviews in high-impact surgical journals. 2017;104(3):198-204. doi: doi:10.1002/bjs.10423.

I have the following discretional comments for the authors:
14. Ll. 60-62: I suggest to consistently use either no decimal or one decimal.

We have now rounded up the figures.

15. Ll. 188: I would reword “inclusion of meta-analyses” to “whether meta-analyses were performed” (and accordingly in other places in the manuscript) to stress that this is an active process.

We have changed the text to “whether meta-analyses were performed” on the list of variables as suggested. We believe “inclusion of meta-analyses” was appropriate for the other places in the manuscript due to its brevity and because it describes objectively whether reviews have included meta-analyses as we saw them.

16. Table 2: There is a space between 52 and % (Searched grey/unpublished literature).

We have deleted the space.

17. Ll. 320: I suggest to reword “35% had at least one study …” to “35% identified at least one study”.

Thanks you. We have changed “had” to “identified” as suggested.

18. Ll. 470: I suggest to delete the word “interestingly”.

We have deleted the word.

**Reviewer 2**

Reviewer #2: The paper is overall well-written, but requires a number of major and minor clarifications in the methods and definitions used, as well as adjusting for multiple comparisons in the analysis. A (very) short summary of the paper would be that it examines whether meta-analyses in the field of Health Services and Delivery Research (HSDR) report examining publiction and outcome reporting bias. This may be helpful to raise awareness of the need to examine these amongst meta-analysts in the HSDR field.

Thank you for your time and informative comments. Please find our responses to each comment below.

Major points:
- In the section 'Sampling Strategy' the authors report that the database healthsystemsevidence.org was used to pre-screen systematic reviews for inclusion, because the scope was too large for manual screening. But that the pre-screening is outsourced in this way does not obviate the need to describe how the corpus was defined. That is, what are the criteria for inclusion of systematic reviews in the healthsystemsevidence.org database? (search phrases etc)

We have briefly described the information sources and key topic areas and scope for the inclusion of systematic reviews in the Heath Systems Evidence database in the ‘Sampling strategy’ section. Further details have been described in a published paper elsewhere (which we have cited when we first mentioned the database [Reference 14, Lavis et al. 2015]) and it is beyond the scope of this paper to inclusion these details.

- The authors run many hypothesis tests (Table 1: 15, Table 2: 9, Table 3: 10, Table 4: 10) and then interpret the p_-values at face value. This overstates the evidence. I suggest correcting for multiple comparisons (e.g., using Bonferroni). Although it can sometimes be difficult to define what a "family" of tests are within which to correct the false-positive rate, in the authors case I think this is fairly straightforward as each table seems to present a different family of analyses (i.e., for Table 15 using a Bonferroni correction, alpha = nominal alpha/15). It is perhaps a little less clear what to do for Table 2:4 where the authors run both a set of univariable analyses and a multivariable analysis, here I would personally correct within each type of analysis. If the authors' are philisophically opposed to multiple comparison corrections for some reason, they need to at the very least interpret their results in view of the many tests they do, although the risk of overstating the evidence is much higher without formal corrections.

Our study is descriptive and the analyses that we undertook were explorative. We thank the reviewer for cautioning our interpretation of findings from these analyses. We took advice from the editor and have removed statistics related to hypothesis testing (i.e. p values) and have taken care to avoid interpreting the findings based on statistical significance.

- Relatedly, the authors seem to largely avoid interpreting their own multivariable regressions? That is:
Line 360-361: "A higher proportion of the intervention review mentioned and assessed both biases compared to the association reviews". Indeed, if we only care about the margins, but if so what were all the regressions about? Main effects are not interesting when we have interactions. It is very strange to ignore the regressions when summarizing the results.

The text referred to here is a brief summary of key findings. We have kept it short and focused on the overall findings related to our stratified sampling strategy. We intentionally omitted results from multivariate regressions here because we were aware of the exploratory nature of the analyses and wanted to avoid over-emphasising and over-interpreting the findings in accordance with the reviewer’s advice.

Line 374: "believe that collinearity is not a major issue judging from the broad consistency between the results of univariable and multivariable analyses". I disagree. For one, many many variables which were "significant" (assuming p <.05) in the univariable version become non-significant in the multivariable regressions. This is even more the case if an adjustment for multiple comparisons had been made. Broad consistency is a very questionable statement here. In addition, Table 1 present evidence *in favor* of collinearity between at least the variable "being an intervention review" and "including a meta-analysis". It is thus unclear whether the comparison between intervention and association reviews is relevant, or if difference are due to including (or not) a meta-analysis in the systematic review.

We have reflected upon the reviewer’s comment, and have revised the text to read as follows: ‘We are aware that the variables which we examined may interact in various ways, as indicated by the changes in estimated ORs between univariable and multivariable analyses for some of the variables examined. The relationship between factors that could impact upon examination of publication and outcome reporting bias in HSDR systematic reviews are intricate and will require further research to clarify.’

-Clarification of why the authors choose to compare "intervention" and "association" reviews, since this affects the presentation of most results. On lines 109-113, the authors state that:
[a] "We hypothesised that association studies may be more susceptible to publication and outcome reporting biases than intervention studies due to the exploratory nature of most association studies."
[b] "We therefore investigate whether the practice of assessing these biases and the findings of these assessments differ beween HSDR systematic reviews focusing on these two types of studies."
However, [b] does not follow from [a]. If the authors' hypothesis is that association studies are more susceptible to publication/outcome bias than intervention studies, then they should surely be researching this question? As it is, the authors presume their hypothesis to be true, and then essentially examine whether systematic reviewers are aware of this "truth". I could just as plausibly argue that, given the authors assumption that association reviews are more exploratory and intervention reviews more confirmatory, intervention studies should be more prone to publication bias and outcome switching because they are more invested in a given outcome than the exploratory association reviews. If so, it would make sense, again according to the authors' line of reasoning, that systematic reviews of intervention studies are more careful to assess potential biases.

We appreciate the reviewer’s observation. Indeed the study reported in this manuscript is part of a larger project which included another study directly investigating hypothesis [a]. For this current study, the presumption that [a] is true did influence our design including making the comparison between intervention and association reviews. This was in part due to pragmatic reasons as we tried to use different approaches to gathering as much information as possible in relation to occurrence and practice concerning publication and outcome reporting bias in HSDR within a single funded research project. We therefore did not have the luxury of sequentially testing hypothesis [a] before designing and undertaking the study reported here. Interestingly, findings from a further component within our wider project that incorporated collection of qualitative evidence from HSDR stakeholders painted a more sophisticated picture, in which different factors either increasing or reducing the occurrence, assessment and potential impact of publication and related bias in HSDR are at play simultaneously, such that different hypotheses may be valid at the same time and need not to be mutually exclusive.


Minor points/clarifications:

*The link to the Warwick Research Archive Portal is missing (currently written as XXXXX)

Thank you, we shall include this before final publication, if the manuscript is accepted.

Line 80: I have not heard of MEDLINE previously and it is presented without an explanation. If this is something extremely well-known in the field of medical research this is fine, but otherwise please provide a short explanation of what it is.

MEDLINE is the prime and most comprehensive database in the field of biomedical and health research, and therefore it is indeed well known in the field.

Line 126: Very nice that the study was pre-registered, but I request a direct link to the pre-registration if possible. Few people check pre-registrations, so they need to be as accesible as possible.

We have included the link www.crd.york.ac.uk/prospero/display_record.php?ID=CRD42016052366

Line 142-143: The categories used from healthsystemsevidence.org are "effectiveness" and "other". A discussion of how well these categories map onto those chosen by the authors(intervention and association) is lacking

We have now added the following text in the Discussion: ‘We targeted intervention and association reviews with a quantitative component in HSDR as defined earlier in this paper. The concept of intervention reviews matches well with the category of ‘systematic reviews of effects’ in the HSE database where we drew our sample. However, clearly delineating association reviews and identifying those incorporating some quantitative components has proven challenging. We had to screening more than a thousand records classified as ‘systematic reviews addressing other questions’ in the HSE to obtain our required sample, as the majority of reviews in this category either adopted descriptive, narrative or qualitative approaches, or did not match our definition of an association review.’

Line 170-171: "such as those investigating effectiveness and cost effectiveness of clinical interventions" -> Please write out fully what was excluded rather that give examples

This sentence refers to what we meant by health technology assessment. We have replaced “such as” with “that is” to make this clearer.

Line 176: "steering committee were consulted when necessary" -> under what circumstances was this necessary?

We have added the follow text: ‘Discrepancies were resolved by discussion and members of the research project management team (in the first instance) and steering committee were consulted when the two authors could not reach an agreement or when generic issues concerning study eligibility criteria were identified.’

Line 187: there should be a reference here for the " minimum number of studies recommended.."

We have now provided the reference.

Line 190: The authors assume " that all Cochrane review adhered to [MECIR] standards even if not reported by authors". However, later the same assumption does not seem to be extended to journals with reporting standards (line 246), this needs to be discussed. In addition, that authors may have used guidelines without reporting their use is an important limitation which is not written out.

We made the assumption for Cochrane reviews as the Cochrane Collaboration has a very stringent editorial process in which compliance with methodological standards are carefully checked by the editorial office before the reviews are published. In contrast, while many journals endorse reporting standards, to our best knowledge these are not necessarily enforced during the peer review and editorial process. We have included the following sentence to address the latter point highlighted in the reviewer’s comment above: “Alternatively, some authors might have used reporting guidelines during the preparation of their reviews without explicitly stating it.’

Line 209: Two coders did the coding, but no agreement level was reported. Please report Cohen's kappa or similar measure of inter-rater agreement.

Data extraction in this study was primarily related to collection of factual information from the sampled systematic reviews. The checking by the second person therefore aimed to identify potential errors and omissions rather than to verify the validity/reliability of coding involving subjective judgement.

AMSTAR: there is a new version of AMSTAR out. I understand the authors used the original measure for consistency with the healthsystemsevidence database, but considering there's an AMSTAR2 version, a critical discussion of the quality of AMSTAR is needed (perhaps in the limitations section). The AMSTAR webpage links several papers that could be a good starting point.

We have included a discussion on limitation of AMSTAR.

Line 227: How do you define "formal" assessment?

We have added the following explanation: ‘….accompanied by formal assessment (with explicitly stated methods, e.g. use of funnel plots or comparison with findings from search of study registries known to capture all related studies that have been conducted; the latter is unlikely to be feasible in HSDR).’

Results: A flowchart indicating the original sample and how many systematic review were excluded and for what reason to reach the final sample would improve clarity

We have now provided the information in a new section ‘Sampling of HSDR systematic reviews from HSE’ at the beginning of the Results section.

Table 5: This table is misplaced. Don't present new information in the discussion section. Move to introduction or methods.Also explain what "NR" stands for.

One of the key functions of the Discussion section is to place the findings of the study within the context of what is already known. We believe the table serves this purpose well in its current place. The information presented in the table was gathered from the literature and so is not new information from the current study. We have added a footnote to indicate that ‘NR’ stands for ‘not reported’.

Line 410-411: "40% of all reviews" in one study is compared with the current study's "10% for association review" -> The appropriate comparison is with % from all review in the current study. Comparing just association reviews is misleading.

We thank the reviewer for pointing out this issue. As the study being referred to in line 410 of our original manuscript focused on intervention studies, we have amended the text to compare the quoted figure with the corresponding figure from our HSDR intervention reviews, and to make the nature of comparisons with other figures from clinical research clearer:

‘For example, we found that a meta-analysis was conducted in only 33% of the HSDR intervention reviews. This is similar to 39% reported in a previous study of Cochrane reviews on health policy interventions [10]. We found an even lower prevalence (10%) of including a meta-analysis in HSDR association reviews. These figures are in contrast with at least 60% observed among both intervention and association reviews in clinical research (Table 5).’

Line 419: "the issue of publication and outcome biases may be perceived as unimportant or irrelevant in review adopting configurative approaches" -> It is suggested a large percentage of HSDR reviews are association reviews that use a configurative approach. This argument would be strengthened by adding what proportion of HSDR reviews are in fact association reviews (or "other", in the healthsystemsevidence database).

We did not claim that a large percentage of HSDR reviews are association reviews. Our statement suggests that HSDR systematic reviews which evaluate intervention effects and associations (particularly the latter) may tend to adopt a configurative approach. This statement is supported by our finding of low and very low prevalence of including meta-analyses in our sampled intervention and association reviews respectively, compared with clinical research as discussed above. We have revised the text so that it is clear that our statement is related to the types (intervention and association) of systematic reviews included in this study.

Line 420: Is there evidence HSDR is *more* diverse and context-specific than the fields that are compared against?

As the reviewer astutely pointed out, it is difficult to say for sure whether HSDR is ‘more’ diverse and context-specific than clinical research, the main field of research which HSDR is often compared and contrasted with. It is beyond the scope of our study to prove this, and we had taken care in our writing not to make such a claim. Nevertheless, the influence of context on findings of HSDR have frequently been highlighted as a major challenge in HSDR, and we have now provided relevant references. We feel it is appropriate to discuss the issue of context and its potential link with the approaches that people may choose to synthesise HSDR evidence in intervention and association reviews.

Line 425-426: Please write out some of these limitations rather than just referencing their existence. Also, to my knowledge these methods do *not* "indicate the presence or absence of publication bias", but of small-study effects.

We have now explained in detail that funnel plots and related statistical techniques can detect small study effects but do not prove the presence or absence of publication bias:

‘Funnel plots and related techniques, the most commonly used methods, require that at least 10 studies of varied sample sizes that are addressing sufficiently similar questions and that have used compatible outcome measures to enable appropriate analyses [35]. In HSDR systematic reviews, the level of heterogeneity among included studies are often high and so reviewers are often not able to use these formal statistical techniques. Irrespective of the technical requirements, such statistical methods could only detect small study effects, which could be suggestive of publication bias but do not prove it, as several potential causes other than publication bias, such as issues related to study design, statistical artefact and chance, could also lead to the presence of small study effects [35]’
